# THE INFLUENCE OF CUSTODIAL STATUS ON MARITAL AFFECTUAL SOLIDARITY AND MENTAL HEALTH AMONG GRANDPARENTS.

**DOI:** 10.1093/geroni/igz038.3433

**Published:** 2019-11-08

**Authors:** Eunbea Kim, Danielle K Nadorff, Rachel Scott, Ian T McKay

**Affiliations:** 1 Mississippi State University, Mississippi State, United States; 2 Mississippi State University, Starkville, Mississippi, United States; 3 Mississippi State, Mississippi State, United States

## Abstract

Increased life expectancy and the diversity of family structure have resulted in a substantial rise in the number of families with grandparents as the main caregivers (e.g. custodial grandparents). The structures of these families affect the well-being of all family members. After middle age, psychological well-being is associated with marital relationship quality, and raising one’s grandchildren is a known source of strain to relationships. The current study examined adults aged 40 and older (M age = 57.6 yr, 53% female) using a nationwide sample from MIDUS to assess the extent to which custodial grandparenting status influences marital affectual solidarity, depressive symptoms, life satisfaction, and perceived stress. Measures included the Center for Epidemiological Studies Depression Index, Spousal Affectual Solidarity, Satisfaction with Life Scale, and Perceived Stress Scale. Marital affectual solidarity was significantly related to custodial status and psychological well-being, and there were significant differences in marital relationship quality and psychological well-being between custodial grandparents and non-custodial grandparents. However, custodial status failed to moderate the relation between marital affectual solidarity and mental health. Although other factors surrounding custodial grandparents likely affect their marital relationship and mental health, these results suggest that grandparents raising grandchildren are under particular strain in their marriages and are in need of targeted interventions to ameliorate stress and depressive symptoms. These findings will inform the need for more research and supportive educational programs on family relationships and the psychological health of custodial grandparents.

